# A Diagnostic Dilemma: Proteinase-3 (PR3)-Positive Anti-neutrophil Cytoplasmic Autoantibodies in Eosinophilic Granulomatosis With Polyangiitis

**DOI:** 10.7759/cureus.80365

**Published:** 2025-03-10

**Authors:** Samuel Goldman, Benjamin Wilson, Kanwal Awan, Tara White, Matthias Williams

**Affiliations:** 1 Internal Medicine, Walter Reed National Military Medical Center, Bethesda, USA; 2 Pulmonary Medicine, Walter Reed National Military Medical Center, Bethesda, USA

**Keywords:** alveolar eosinophilia, anca associated vasculitis, asthma, broncho-alveolar lavage, eosinophilic granulomatosis with polyangiitis (egpa), mepolizumab, nonallergic rhinitis, pancreatitis causes

## Abstract

Eosinophilic granulomatosis with polyangiitis (EGPA) is a multifaceted diseased vasculitis typically associated with myeloperoxidase-perinuclear-anti-neutrophil cytoplasmic antibody (+MPO-P-ANCA). Although rare, the diagnosis should be considered in patients with difficult-to-control or late-onset asthma and extrathoracic disease. We present the case of a 37-year-old male with adult-onset asthma and chronic rhinitis hospitalized with pancreatitis and hypoxemia. Blood investigations demonstrated eosinophilia with elevated lipase, and bronchoscopy demonstrated multiple endobronchial lesions with elevated eosinophils on bronchoalveolar lavage. In addition, labs showed proteinase 3-specific antineutrophil cytoplasmic antibody (PR3-C-ANCA) autoantibodies, and the patient was diagnosed with EGPA. Additional differential diagnoses of parasitic infection, sarcoidosis, chronic eosinophilic pneumonia, and granulomatosis with polyangiitis (GPA) were entertained, but ultimately, the multisystemic involvement, anti-neutrophil cytoplasmic autoantibody (ANCA) positivity, bronchoscopy, and imaging findings clinched the diagnosis. This case highlights the spectrum of possible EGPA presentations and a rare case of PR3-C-ANCA with gastrointestinal manifestations.

## Introduction

Eosinophilic granulomatosis with polyangiitis (EGPA) is a challenging clinical diagnosis due to the heterogeneity of symptoms and low incidence. EGPA is one of the least common causes of anti-neutrophil cytoplasmic autoantibody (ANCA) associated vasculitis, with an estimated incidence of four cases per million and a prevalence of 18 cases per million [1,2]. Disease classification is broad, and the American College of Rheumatology (ACR) defines the clinical and laboratory criteria for the diagnosis of EGPA. If a patient displays a combination of clinical and laboratory criteria, including obstructive airway disease, nasal polyps, mononeuritis multiplex, peripheral eosinophilia, and inflammatory eosinophilia on tissue biopsy, they meet the diagnosis for EGPA [3]. Even with diagnostic criteria, the different phases of EGPA presentation, in addition to two primary subtypes of EGPA, can complicate diagnosis.

The three primary phases include the allergic phase, the eosinophilic phase, and the vasculitic phase. The first phase is marked by prominent respiratory tract involvement, usually asthma and sinus disease. The eosinophilic phase progresses with eosinophilic infiltration of organ systems, including the lungs, heart, kidneys, and gastrointestinal tract. The third and final phase demonstrates necrotizing vasculitis of the involved systems [4]. Additionally, two subtypes of EGPA predominate: a vasculitic ANCA positive subtype characterized by glomerulonephritis, purpura, and mononeuritis and an eosinophilic ANCA negative subtype characterized by cardiomyopathy and lung infiltrates [5].

Despite these well-documented classification systems, up to 20-40% of cases have some degree of overlapping or atypical features, such as gastrointestinal abnormalities (such as pancreatitis). Furthermore, EGPA patients with proteinase-3 (PR3)-positive ANCA present this atypical phenotype compared to either the vasculitic or eosinophilic subtypes.

## Case presentation

A 37-year-old active-duty male with a past medical history significant for asthma and pancreatitis presented to the emergency department with progressive dyspnea on exertion and epigastric pain, as well as new onset scant hemoptysis and sinus discomfort. Dyspnea on exertion and epigastric pain were present for 15 months prior to presentation, with the former refractory to outpatient management with fluticasone propionate (0.11 mg/actuator, two puffs inhaled twice daily), albuterol (0.9 mg/actuator, two puffs inhaled every four hours as needed), and fluticasone/salmeterol (100mcg-50mcg, one puff inhaled twice daily). The patient’s asthma was diagnosed four months prior to presentation via a methacholine challenge test. The structural evaluation at that time with chest radiography was unremarkable. Regarding his pancreatitis, he previously underwent abdominal computed tomography (CT) and magnetic resonance cholangiopancreatography (MRCP) without evidence of pancreatic, gallbladder, or biliary tree abnormality. Despite this extensive evaluation, the patient had persistent symptoms prior to the emergency department presentation.

**Figure 1:**
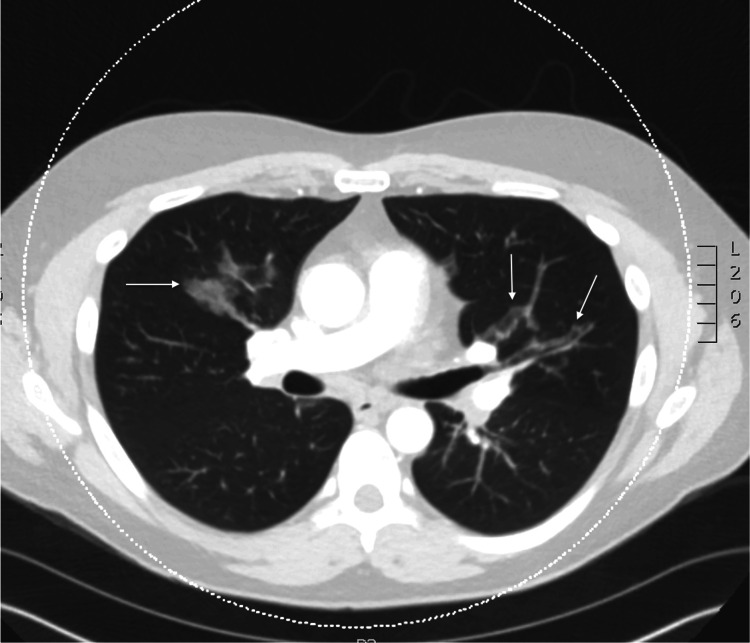
Axial view of non-contrasted chest CT demonstrating multifocal, predominantly peribronchial ground glass opacities (arrows).

**Figure 2:**
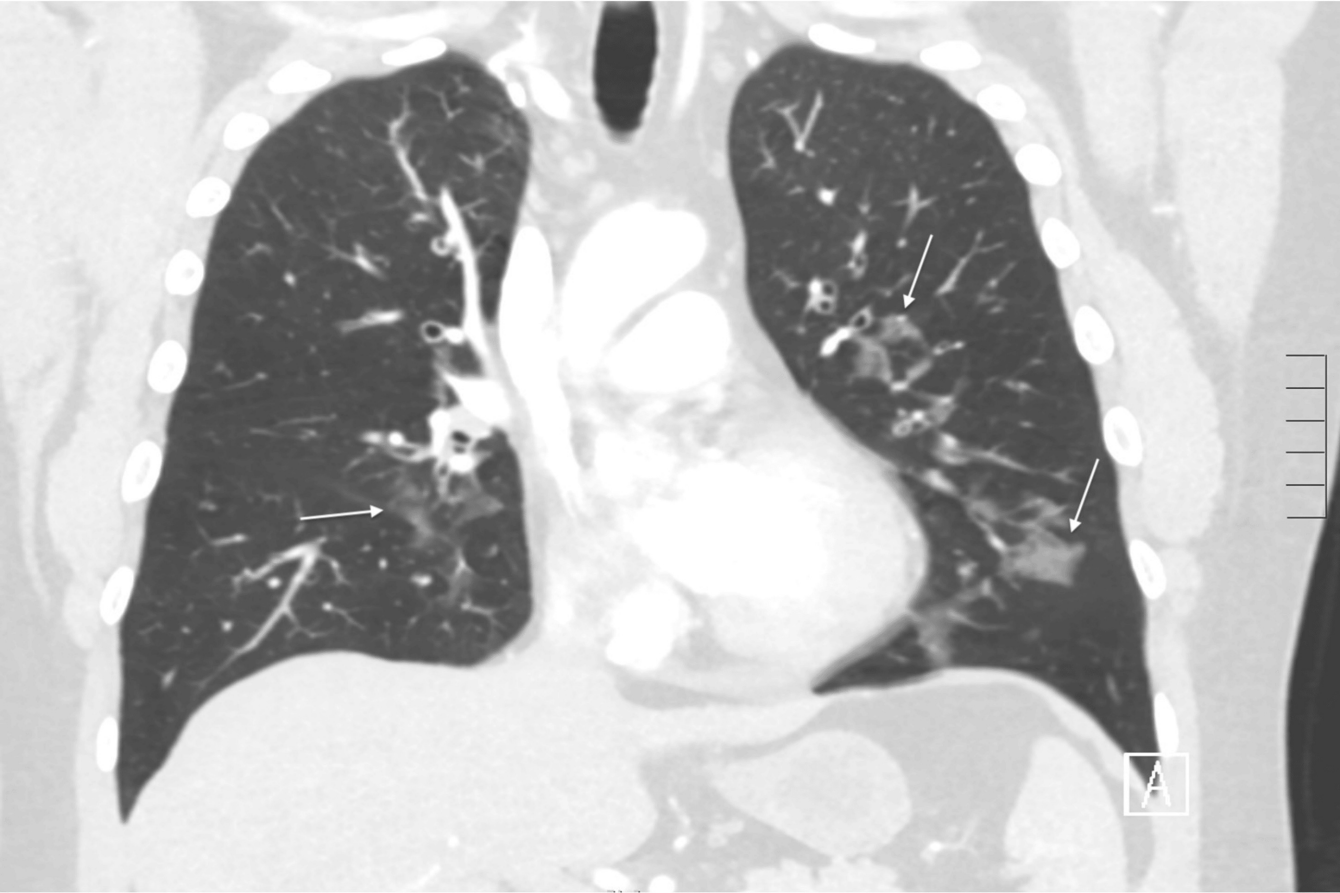
Coronal view of non-contrasted chest CT demonstrating multifocal, predominantly peribronchial ground glass opacities (arrows).

**Figure 3:**
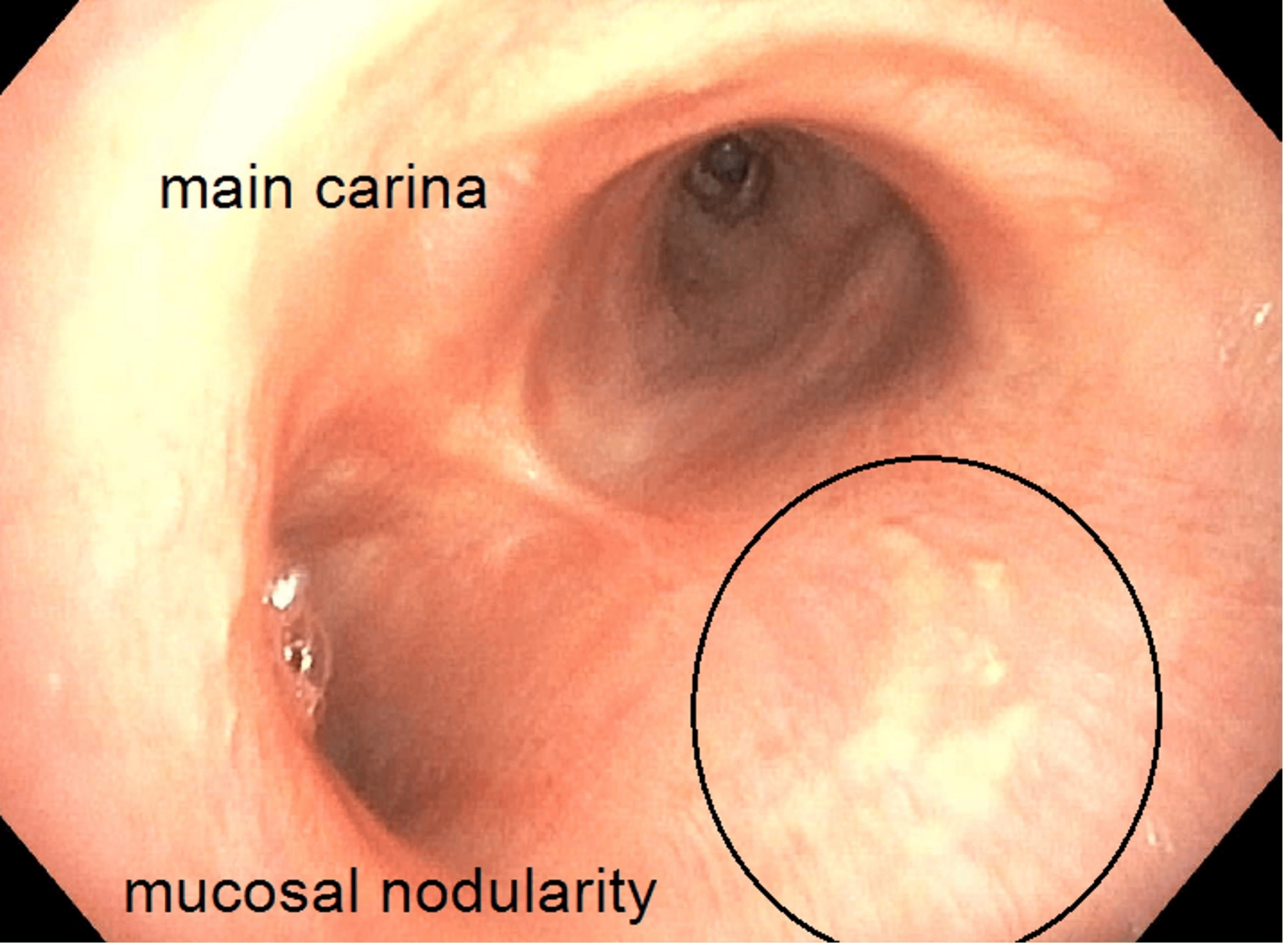
Bronchoscopy demonstrating mucosal nodularity (circled)

**Figure 4:**
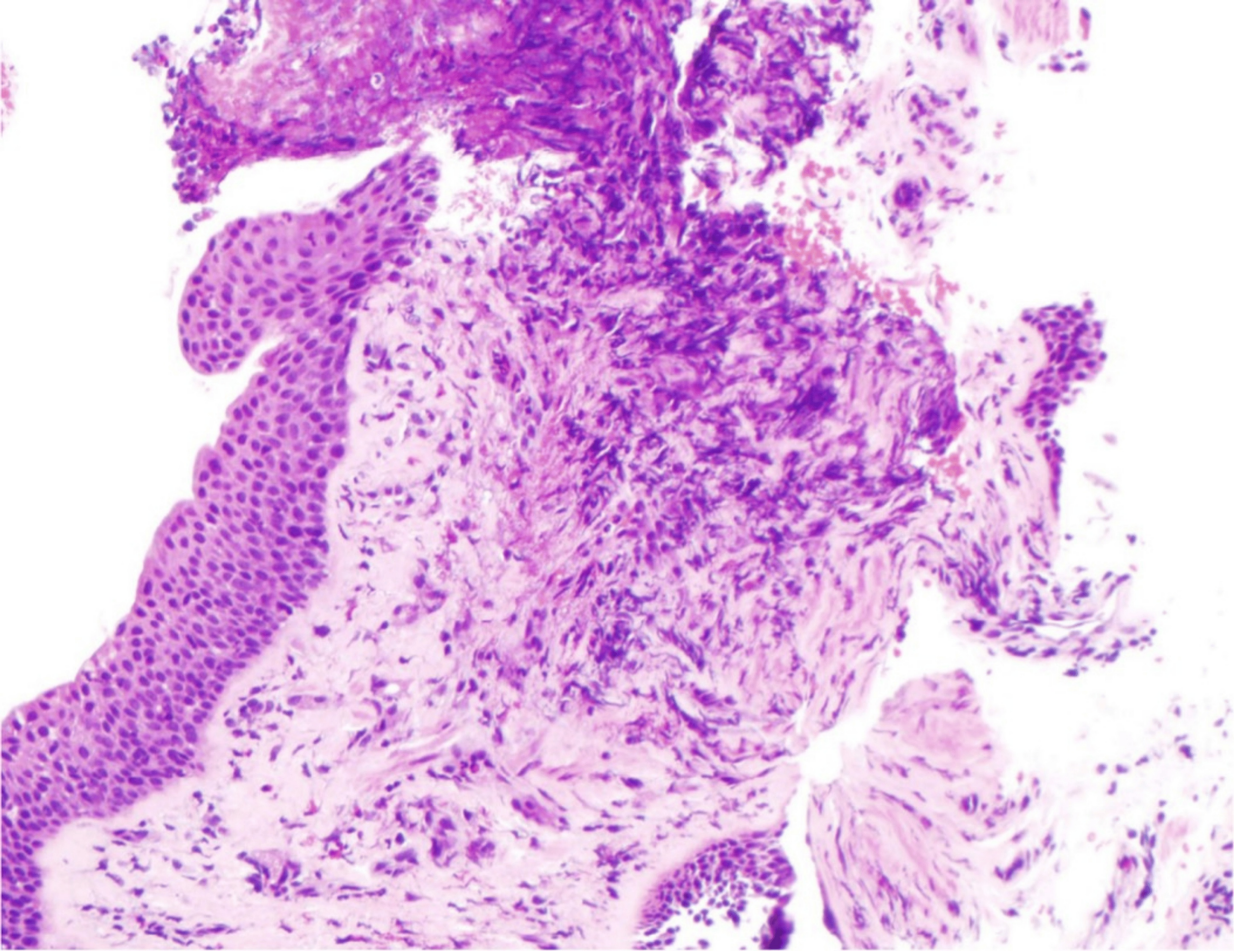
Forceps biopsy demonstrating eosinophilic infiltrate (Hematoxylin and Eosin stain; 20x magnification, low-power light microscopy)

In the emergency room, the patient was found to be tachypneic (33 respirations/minute), tachycardic (109 beats per minute), hypertensive (150 mmHg systolic/90 mmHg diastolic), and hypoxemic (92% via pulse oximeter while on two liters nasal cannula). The patient was afebrile. The physical exam was notable for abdominal tenderness in the left upper quadrant and epigastric region, and the patient was noted to have diffused wheezing in all lung fields. Admission labs demonstrated a leukocytosis (WBC count of 14.6 cells/mcl (normal 4.2-9.2 cells/mcL)), with an elevated eosinophil count (2.66 x10^3^ cells/mcL (normal <0.3 x10^3^ cells/mcL)) and an elevated lipase (202 U/L (normal 13-60 U/L)). A preliminary infectious workup, including HIV and toxocariasis, was negative. A chest computed tomography scan with contrast demonstrated new multifocal ground glass opacities and mediastinal lymphadenopathy (Figures 1-2). Due to imaging findings, he underwent a bronchoscopy, which demonstrated inflammatory changes and mucosal nodularity (Figure 3). Bronchoalveolar lavage revealed no infectious process with negative fungal studies, negative acid-fast bacilli smear, and negative bacterial cultures. Forceps biopsy from the trachea, right upper lobe, and left lower lobe demonstrated squamous metaplasia with a dense eosinophilic infiltrate (Figure 4). No infectious or fungal elements were seen. Additional lab tests showed an elevated IgE level (493.50 IU/mL (normal 0-100 IU/mL)) and an elevated proteinase 3 antibody (5.5 U/mL (normal 0-3.5 U/mL)). A subsequent sinus computed tomography scan showed bilateral paranasal sinus disease and acute sinusitis.

During the initial investigation, the patient received treatment for asthma exacerbation due to suspected underlying community-acquired pneumonia with ceftriaxone (1g intravenous daily) and azithromycin (500 mg intravenous daily). With the obstructive airway disease, sinus disease, peripheral eosinophilia, and extravascular eosinophilic inflammation on biopsy, the patient was diagnosed with EGPA. Following this diagnosis, the patient was started on prednisone 40mg daily. The patient’s hypoxemia was resolved, and the antibiotics were discontinued following bronchoscopy. The patient was discharged from the hospital with a prednisone taper. He was referred to rheumatology for the initiation of anti-interleukin-5 therapy (Mepolizumab) and started on therapy one month post-hospitalization with substantial improvement in symptoms.

## Discussion

Our patient’s case demonstrates the complexity of making a firm diagnosis of EGPA. Even with his positive PR3+ ANCA serology, he still met the diagnostic criteria [3], and the uniqueness of his case required careful consideration of other causes. The initial differential included viral pneumonia, parasitic or fungal infection, sarcoidosis, hypersensitivity pneumonitis, chronic eosinophilic pneumonia, and granulomatosis with polyangiitis (GPA), in addition to the eventual diagnosis of EGPA.

Viral etiologies were quickly excluded from the differential, given the negative respiratory viral polymerase chain reaction (PCR). The patient had no risk factors for or known exposure to parasitic infections, making parasitic pneumonia unlikely. The 2015 EGPA Consensus Task Force suggests evaluating for atypical infections, familial, and paraneoplastic causes when diagnosing EGPA [6]. The patient’s initial panel of labs included negative HIV and toxocariasis serum testing, in addition to negative fungal cultures on bronchoalveolar lavage. Aspergillus antibodies on post-hospital follow-up were also negative.

Sarcoidosis was considered, given multi-organ involvement, hypoxemia, and lymphadenopathy on chest computed tomography prior to presentation. However, pancreatic sarcoid typically presents as a mass lesion [7], and our patient had prior abdominal CT and MRCP without evidence of pancreatic mass. Additionally, while mediastinal lymphadenopathy can be a finding in sarcoidosis, pulmonary nodularity is typically in a peribronchovascular distribution, unlike the tracheal nodularity seen in this case. 

Chronic eosinophilic pneumonia (CEP) and hypersensitivity pneumonitis both have some overlap with this case’s clinical presentation. In particular, the incidence of asthma in over 2/3rds of chronic eosinophilic pneumonia patients would fit with the patient’s presentation [8]. However, extra-thoracic manifestations do not occur with CEP and favor the diagnosis of EGPA [9]. The patient was without associated exposures or changes in an occupational setting to suggest hypersensitivity pneumonitis, and hypersensitivity pneumonitis typically has a lymphocytic predominant infiltrate, which did not fit the eosinophilic predominance seen in this case [10].

Finally, we considered GPA on our differential due to the patient’s PR3+ ANCA in addition to his nasal and pulmonary involvement and scant hemoptysis. Although the vessel size and organ system involvement are similar, findings on pathology with GPA demonstrate necrotizing granulomas and typically lack eosinophilic predominance. Lung findings can involve cavitary nodules, which were absent in this case. Furthermore, renal involvement occurs in up to 70% of cases with a rapidly progressive glomerulonephritis in up to 50% of cases [11], which was absent in our patient.

Regarding the patient’s clinical course, he was likely in the first and second phases of the disease process at the time of presentation as he demonstrated symptoms consistent with the allergic phase (asthma, sinusitis) and the eosinophilic phase (eosinophils, radiographic opacities, pancreatitis), but did not yet demonstrate signs of the vasculitic phase (no necrotizing granulomas on biopsy) [2]. This means his EGPA was identified correctly, and the appropriate treatments were started before he progressed to the later stages of the illness.

The cause of EGPA remains incompletely understood. It is considered idiopathic, but Th2, Th1, and Th17 T-cells are thought to be major mediators of the disease, and development of ANCA (in 30-40% of cases), upregulation of IgE and interleukin-5, and eosinophil activation are likewise contributing factors. However, the connection between ANCA and eosinophils has not been established [12], and a full understanding of the pathophysiology has not yet been elucidated. These mechanisms are involved in both the eosinophilic and vasculitic subtypes, and ANCA is most associated with the latter. Even though our patient was ANCA-positive, his pulmonary infiltrates and gastrointestinal involvement are more common in ANCA-negative disease [5]. Notably, he did not develop renal disease at any point in his disease course, which is more closely linked to ANCA-positive phenotypes [5,13].

It is also unique that our patient was PR3-ANCA positive, as this is very rarely associated with EGPA [14] and counts against the most recent diagnostic criteria [3]. PR3-ANCA EGPA has been associated with less frequent incidence of asthma, lower eosinophil counts, and more frequent vasculitis relapses as compared to myeloperoxidase (MPO)-ANCA or ANCA negative EGPA patients [15], but our patient still expressed a clinical course largely consistent with MPO-ANCA and ANCA-negative EGPA. Like PR3-ANCA, pancreatitis is also very uncommon. Roughly 25% of ANCA-negative EGPA and 20-40% of ANCA-positive EGPA will have gastrointestinal involvement [5], but pancreatitis may only represent 5% of gastrointestinal complaints in any ANCA-associated vasculitis and is very rarely reported with EGPA [16]. Other case reports have described similarly rare variations of EGPA, such as rhabdomyolysis and MPO-ANCA positive serology [4] or myopericarditis as a presenting symptom [17], but this case represents a unique pattern of findings.

## Conclusions

This case highlights the diagnostic challenges that accompany highly variable, systemic diseases such as EGPA. While our patient had several classic features and was ultimately given the correct diagnosis, non-classic findings meant alternative diagnoses had to be very carefully considered and ruled out. Other case reports have described similarly rare variations of EGPA, such as rhabdomyolysis and MPO-ANCA positive serology or myopericarditis as a presenting symptom, but this case represents a unique constellation of symptoms and findings. It is critical to maintain a focused differential during the diagnostic workup and consider unusual findings as possible manifestations of EGPA.
